# Patient Perception of University Hospitals' PCs for People Initiative

**DOI:** 10.7759/cureus.86355

**Published:** 2025-06-19

**Authors:** Paige E Harbarger, Claudia I Cabrera, Brian D'Anza

**Affiliations:** 1 Otolaryngology - Ear, Nose, and Throat, University Hospitals Cleveland Medical Center, Cleveland, USA; 2 Otolaryngology - Head and Neck Surgery, Center for Clinical Research, University Hospitals Cleveland Medical Center, Cleveland, USA; 3 Otolaryngology - Head and Neck Surgery, University Hospitals Cleveland Medical Center, Cleveland, USA; 4 Digital Health, University Hospitals Health System, Cleveland, USA

**Keywords:** digital divide, low socioeconomic status (ses), medicaid population, quality of life, social and community works, technology gap

## Abstract

The COVID-19 pandemic and post-pandemic world have highlighted the integral part that social connections play in one's mental and emotional well-being. The modern world had to suddenly adapt and accommodate an abundantly virtual one. Those who were unable to make these technological adaptations, many being low socioeconomic households, were facing many new obstacles, such as losing in-person jobs and being disconnected from their community and services by not having the technology to stay in contact, all while living through a global pandemic. University Hospitals understands and recognizes the value of connectivity for lower-income households, and through their Rainbow Connect program, they have partnered with a non-profit technology distribution service to provide a laptop computer and six-month prepaid access to a broadband internet receiver to Medicaid patients. The aim of this study is to evaluate the impact of this initiative on the lives of its recipients. Through an attitudinal survey administered on the day they received their computer and internet hotspot and again after six months, researchers investigated the change in perspective the 100 participants had about owning a PC and what it could do for their lives. This study found significant improvement in participants' perceived mental, emotional, and physical well-being. Participants reported feeling a greater sense of connection to friends, family, and their community. They felt their health was at less risk by being able to stay home for health appointments and by ordering goods and services to their home. They also revealed new avenues for work, as those who have difficulty leaving the house now have the ability to work remotely and attain an income. These results are important in highlighting the changes in recipient perception and encouraging the expansion of similar programs that aim to bridge the digital access gap.

## Introduction

Connectivity to friends, family, and community is an integral part of one's mental [1] and emotional well-being [2]. This has been especially notable over the last few years, during the ongoing COVID-19 pandemic and the ensuing mandated lockdowns and social distancing measures that separated individuals from their social networks and support [3]. The modern world had to adapt and accommodate an abundantly virtual one, as digital communication has been used in the past to bridge gaps in connection when physical connection is not available, as seen in senior living homes being equipped with tablets and the internet for their residents to message and video chat with friends and family members on the outside [4,5]. Many systems in government, medicine, and education have had to be overhauled to remain effective and serve their communities. Those who were unable to make these adaptations alongside the rest of the world faced many new obstacles alongside a global pandemic [6,7]. Many low-socioeconomic households struggled to find affordable technology and internet access, hindering their participation in this technological evolution [8].

In early 2021, 27% of adults living in households earning less than $30,000 a year were smartphone-only internet users, meaning they owned a smartphone but did not have broadband internet at home [9]. This leaves many households trying to accommodate tasks like work, schoolwork, seeking government assistance, and applying for jobs on a mobile device. This growing dependence on the internet and equipped technology to receive services and communicate with others highlights the many obstacles low-income households have while attempting to work on a small-screen smartphone. This is commonly referred to as the "digital divide" [10], and the benefits some communities enjoy from having larger-screen devices, such as computers, and readily available broadband internet for the household rather than just an individual device.

There is evidence that this disparity in technology further extends to race, with reportedly 80% of White adults owning a desktop or laptop computer, compared with 69% of Black adults and 67% of Hispanic adults. Furthermore, 80% of White adults also report having a broadband connection at home, while smaller shares of Black and Hispanic adults report the same, 71% and 65%, respectively [11]. For much of the history of the United States, it has been well known that Black communities have been historically marginalized and overrepresented in socioeconomic measures of health and wealth. In fact, in 2022, Black individuals made up 20.1% of the population in poverty, but were only 13.5% of the population. Hispanics were also overrepresented, exhibiting a higher ratio of impoverished individuals compared to their overall population [11]. It is notable that while Black and Hispanic populations have higher rates of poverty, they have lower rates of computer ownership and broadband internet access. One of the purposes of this study was to explore the sentiments of a disadvantaged population regarding the benefits of having a computer and an internet connection. We compared the perceived benefits of computer access and broadband internet connection, both before and after individuals received them.

These types of technological barriers have inspired programs that provide free or subsidized internet and internet-connected devices to low-income individuals. One example is the "PCs for People" initiative, a national non-profit organization that works to provide low-cost, high-quality computers and internet access to individuals, families, and communities who are historically marginalized and have low incomes. By recycling and then refurbishing computers, these non-profits offer a valuable service to businesses, families, and the planet, keeping computers out of landfills and repurposing them to promote digital inclusion [12]. The hope is that having this connection to the internet allows individuals the opportunity to receive and share vital information, communicate with friends, family, and their community, and access healthcare through tele-health services. Additionally, non-profits such as "PCs for People" aim to enable individuals who may lack a computer or reliable internet connection to engage more fully in the economy by utilizing online ordering, delivery services, and remote work opportunities. While all of these benefits offer great promise, there is a lack of scientific research on the recipients' perception of technology and how this might change before and after such a program.

University Hospitals understands and sees the value in this connectivity, particularly for those lower-income households. Through their Rainbow Connect program, a program designed to "identify and help meet families' basic social needs, things like food, clothing, diapers, utilities, and furniture, to help them stay healthy," they have partnered with "PCs for People" to help provide laptops and internet receivers to patients in need [13]. The current study aims to evaluate the impact of this initiative on the lives of the participating computer recipients. Through an attitudinal survey conducted on the day participants received their computer and internet hotspot, and subsequently at the six-month interval, researchers investigated the changes in perspective participants had about owning a PC and what it could do for their lives.

## Materials and methods

Participants

Participants were 100 subjects recruited via their Medicaid status within University Hospital's patient network in Cleveland, Ohio. The inclusion criteria for this study were as follows: participants were required to be 18 years of age or older and had received a computer as part of the Rainbow Connect initiative. Each participant was provided with a computer and six months of paid broadband internet access as part of a joint initiative between Rainbow Connect and PCs for People. They were made aware that after six months, they could continue internet access for a reduced fee via PCs for People.

After receiving their PC and broadband internet receiver, the subjects were approached about the study. Those who expressed interest were given written informed consent, and after obtaining their signature, they were enrolled in the study. Co-investigators and study coordinators explained the informed consent form to the participants. The study was explained verbally, providing all pertinent information (purpose, procedures, risks, benefits, alternatives to participation, etc.), and ample time was given for questions and concerns to be addressed.

A potential benefit of participating in this study may be to society. By allowing for investigations of initiatives like the current study, more information about the impact these types of initiatives may have on the quality of life perceptions of participants may be discovered, which in turn may encourage other institutions to adopt similar programs. A direct benefit of participating in the study was that participants were also compensated with $10 gift cards for subsequent surveys. Receiving a PC was part of the inclusion criteria, but the survey and computer distribution were conducted independently. The researchers were not directly affiliated with PCs for People. Confidentiality was the only perceived risk to participants, as someone not approved on the study team may breach a participant's personal data. This risk was mitigated by limiting access to study materials to a select number of people and by storing all data behind firewalls on password-protected devices and sites. Subjects had the right to withdraw from the study at any point and for any reason, and they could maintain their computers and internet devices. Subsequently, of the 100 participants who completed the baseline study, 50 participants continued their enrollment in the study by responding to at least one of the follow-up surveys.

Study design

This is a descriptive, longitudinal, prospective study that recruited subjects from individuals who received a computer as part of the joint initiative between the University Hospitals Rainbow Connect Program and PCs for People (a nonprofit organization providing refurbished computers to low-income individuals). This study was approved by the University Hospitals IRB on July 23, 2022, and went into effect the same day (approval number: STUDY20210906). After the individuals who expressed interest in the study provided informed consent and were enrolled, they completed an initial baseline survey that captured their demographic information, computer literacy skills, and perceptions of technology-related quality of life.

The survey instrument was custom-developed by the research team to gather descriptive insights relevant to the study's objectives, rather than to generate quantitative scores or measure objective constructs. The questionnaire design was informed by existing digital literacy assessment frameworks in the literature [14,15]. Although it was not formally validated or pre-tested, it was adapted to the target population and aligned with the study's research aims (Appendices, Supplemental Materials 1, 2).

At one, three, and six months after the initial baseline survey, a follow-up survey was sent via a Health Insurance Portability and Accountability Act (HIPAA)-compliant Research Electronic Data Capture (REDCap) software or conducted by phone to evaluate their perspective on having a PC and internet access at home. For participants reached by phone, responses were entered directly into REDCap by the research team. A limited number of subjects completed the one- and three-month surveys, with only 10 participants having completed the one-month survey and seven having completed the three-month survey; the one-month data points were excluded, focusing on the six-month and three-month data points, which resulted in 50 data points for each of the 100 participants who enrolled in the study and completed the initial survey (50% retention rate). Researchers evaluated any median changes through Likert scores. Patient-specific identifying results, such as names, addresses, survey answers, and other personal information, were not shared with the participants or their healthcare providers, maintaining data confidentiality in accordance with privacy regulations and ethical research practices.

Descriptive statistics were employed to provide a comprehensive summary of the survey data, tailored to the nature of each variable. For categorical variables, such as gender and race, frequencies and percentages were calculated to illustrate the distribution of responses across different categories. For continuous variables, the approach depended on the distribution of the data. If the data were normally distributed, the mean and standard deviation (SD) were calculated to provide an average value and a measure of variation, respectively. Household size data were standardized by recoding the "More than 10" category to a numeric value of 10 (n=1), ensuring that the variable was treated as a continuous numeric measure for subsequent analysis. The IRB evaluated the study and determined that it was exempt. For the non-normally distributed data, the median and interquartile range (IQR) were used to summarize the central tendency and dispersion.

## Results

**Figure 1:**
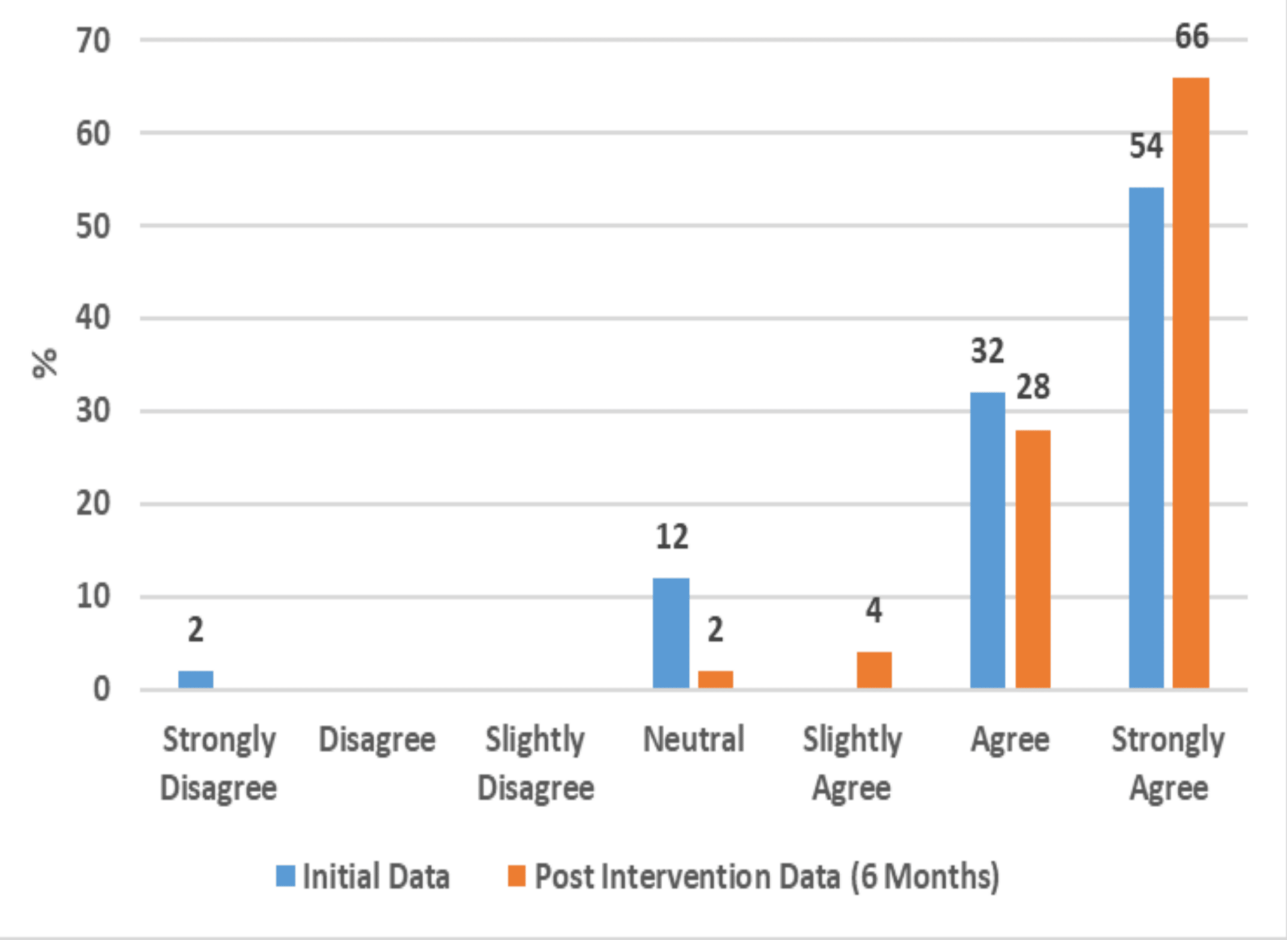
Perception of the Importance of Computer Ownership (50 Respondents)

Given the exclusion of data sets for non-responses, this study focused on the six-month data set, as this group had the highest response rate (50%), and evaluated the responses of those 50 respondents out of the 100 participants (Table 1) (Supplemental Material 3). This study found that, by the six-month survey, participants' perceptions of computers had changed positively in many ways (Supplemental Material 4); 66% of participants strongly agreed and 28% agreed that it was important to own a computer (Figure 1).

**Table 1:** Population Demographics at Baseline

Questions	Levels	Overall, n = 50
What is your gender? (%)	Female	49 (98.0)
	Male	1 (2.0)
What is your age? (median (IQR))	-	31.00 (26.25, 37.75)
What is your race? (%)	Black/African American	41 (82.0)
	Other	6 (12.0)
	White/Caucasian	3 (6.0)
What is your highest level of education? (%)	Associate's degree	8 (16.0)
	High school diploma or GED	38 (76.0)
	Less than a high school diploma	4 (8.0)
What is your household income range? (%)	$20,000 - $44,999	10 (20.0)
	$45,000 - $139,999	1 (2.0)
	Less than $20,000	39 (78.0)
Is this a single household income? (%)	No	5 (10.0)
	Yes	45 (90.0)
How many individuals are in your household? (median (IQR))	-	3.00 (2.00, 4.00)
How many of those individuals are children under 18 in your household? (median (IQR))	-	2.00 (1.00, 3.00)
What is your primary language spoken at home? (%)	English	50 (100.0)
Do you speak another language at home (%)	None (No other language spoken)	46 (92.0)
	Spanish	4 (8.0)
County of residence? (%)	Ashtabula County	1 (2.0)
	Cuyahoga County	47 (94.0)
	Geauga County	1 (2.0)
	Summit County	1 (2.0)
Have you ever used a computer? (%)	No	3 (6.0)
	Yes	47 (94.0)
Why was it hard or difficult for you to get a computer before today? (%)	Didn't know if I would need or use a computer	3 (6.0)
	Didn't know which computer to buy	3 (6.0)
	It costs too much	37 (74.0)
	No internet access	2 (4.0)
	Other	5 (10.0)
Other barriers, please explain. (%) (free text)	It cost too much, couldn't connect to wifi	1 (20.0)
	Need another computer for child use; mine's not working properly	1 (20.0)
	No problem at all	1 (20.0)
	Not difficult	1 (20.0)
	not hard	1 (20.0)
How comfortable do you feel using the computer? (%)	1. Very Uncomfortable	13 (26.0)
	2. Uncomfortable	1 (2.0)
	3. Slightly Uncomfortable	1 (2.0)
	4. Neutral	6 (12.0)
	5. Slightly Comfortable	2 (4.0)
	6. Comfortable	13 (26.0)
	7. Very Comfortable	14 (28.0)
How comfortable do you feel using the computer? (Numeric Likert) (median (IQR))	-	6.00 (1.25, 7.00)
Have you owned a computer before? (%)	No	21 (42.0)
	Yes	29 (58.0)
Do you currently have a computer in your home or a computer that you use at home? (%)	No	45 (90.0)
	Yes	5 (10.0)
If no, is this due to financial reasons or personal preference? (%)	Financial	40 (88.9)
	Preference	5 (11.1)
Do you have access to the internet at home, not by using your cell phone? (%)	No	20 (40.0)
	Yes—Broadband/Highspeed	20 (40.0)
	Yes—Hotspot	7 (14.0)
	Yes—Other	3 (6.0)
Can you access your email at home? (%)	No	6 (12.0)
	Yes	44 (88.0)
Do you have access to the internet or email at home through other means, for example, a smartphone or tablet? (%)	No	6 (12.0)
	Yes	44 (88.0)
Has not having a computer at home made it difficult for you to access health appointments or health information? (%)	1. Strongly Disagree	5 (10.0)
	2. Disagree	6 (12.0)
	3. Slightly Disagree	1 (2.0)
	4. Neutral	15 (30.0)
	5. Slightly Agree	10 (20.0)
	6. Agree	7 (14.0)
	7. Strongly Agree	6 (12.0)
Has not having a computer at home made it difficult for you to communicate with others? (%)	NA	4 (8.0)
	No	27 (54.0)
	Yes	19 (38.0)
Has not having a computer at home made you feel isolated from others? (%)	1. Strongly Disagree; Not feeling isolated at all	6 (12.0)
	2. Disagree	14 (28.0)
	3. Slightly Disagree	3 (6.0)
	4. Neutral	10 (20.0)
	5. Slightly Agree	6 (12.0)
	6. Agree	7 (14.0)
	7. Strongly Agree; Feeling Strongly Isolated	4 (8.0)
Do you feel as though you have missed opportunities due to not having a computer? (%)	1. Strongly Disagree	3 (6.0)
	2. Disagree	3 (6.0)
	3. Slightly Disagree	1 (2.0)
	4. Neutral	6 (12.0)
	5. Slightly Agree	11 (22.0)
	6. Agree	14 (28.0)
	7. Strongly Agree	12 (24.0)
Do you feel as though this computer will help you connect to others? (%)	1. Strongly Disagree	2 (4.0)
	4. Neutral	6 (12.0)
	5. Slightly Agree	5 (10.0)
	6. Agree	24 (48.0)
	7. Strongly Agree	13 (26.0)
Do you feel it is important to own a computer? (%)	1. Strongly Disagree	1 (2.0)
	4. Neutral	6 (12.0)
	6. Agree	16 (32.0)
	7. Strongly Agree	27 (54.0)
Do you feel your health was at risk by having to leave your home due to not having a computer to be able to order goods and services sent to your home during the COVID-19 pandemic quarantine mandates (for example, groceries, clothing, appointments, etc.)? (%)	1. Strongly Disagree	5 (10.0)
	2. Disagree	10 (20.0)
	3. Slightly Disagree	1 (2.0)
	4. Neutral	8 (16.0)
	5. Slightly Agree	5 (10.0)
	6. Agree	12 (24.0)
	7. Strongly Agree	9 (18.0)
Do you feel that having a computer at home will enhance your life? (%)	4. Neutral	10 (20.0)
	5. Slightly Agree	3 (6.0)
	6. Agree	18 (36.0)
	7. Strongly Agree	19 (38.0)

**Figure 2:**
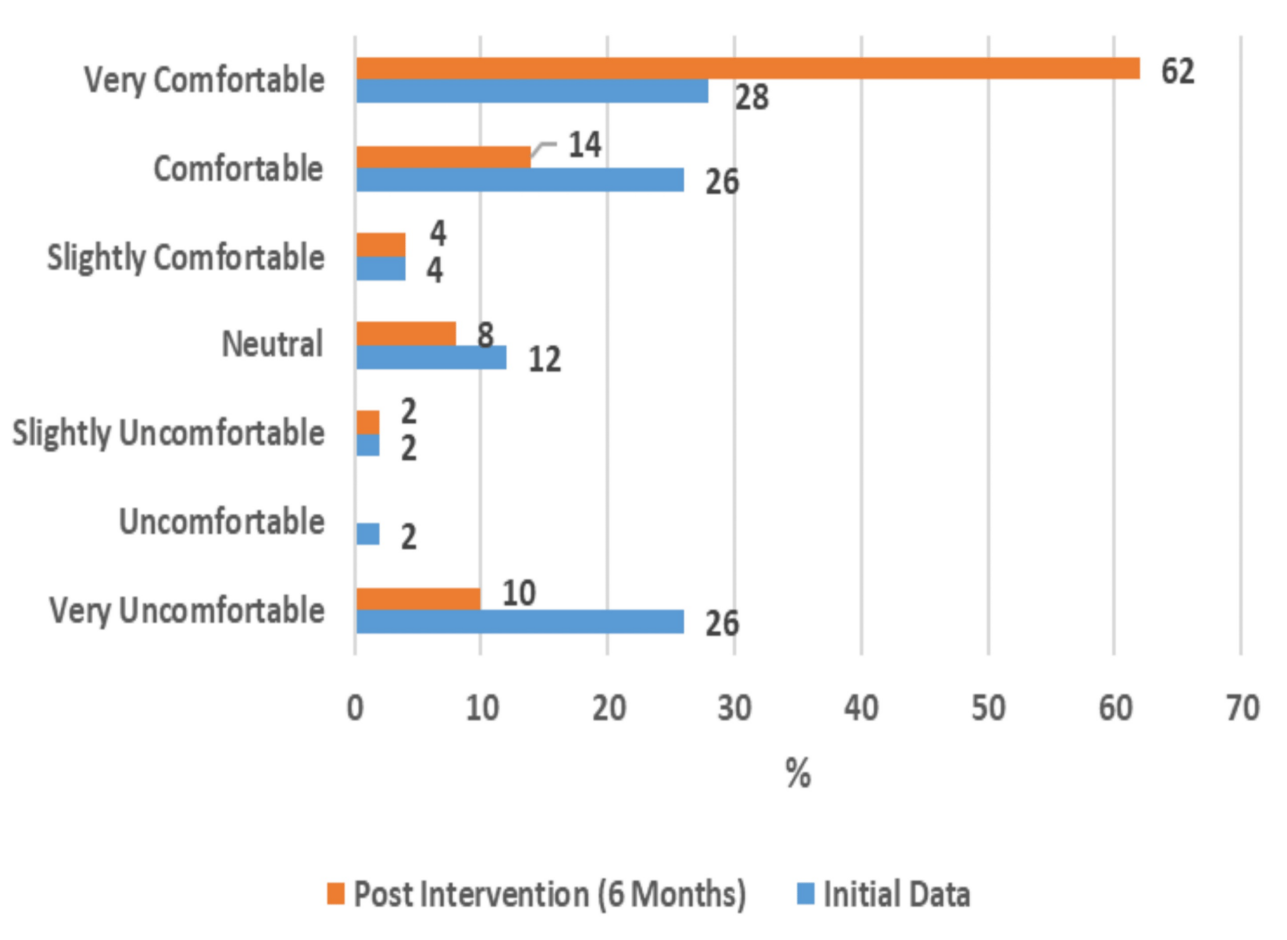
Feelings of Comfort Using a Computer (50 Respondents)

Furthermore, 62% of the subjects reported being very comfortable and 14% reported being comfortable with their computers (Figure 2). This comfort level increased by 34% from the initial survey to the six-month survey. Changes in comfort can be found in the Appendices (Supplemental Material 5).

**Figure 3:**
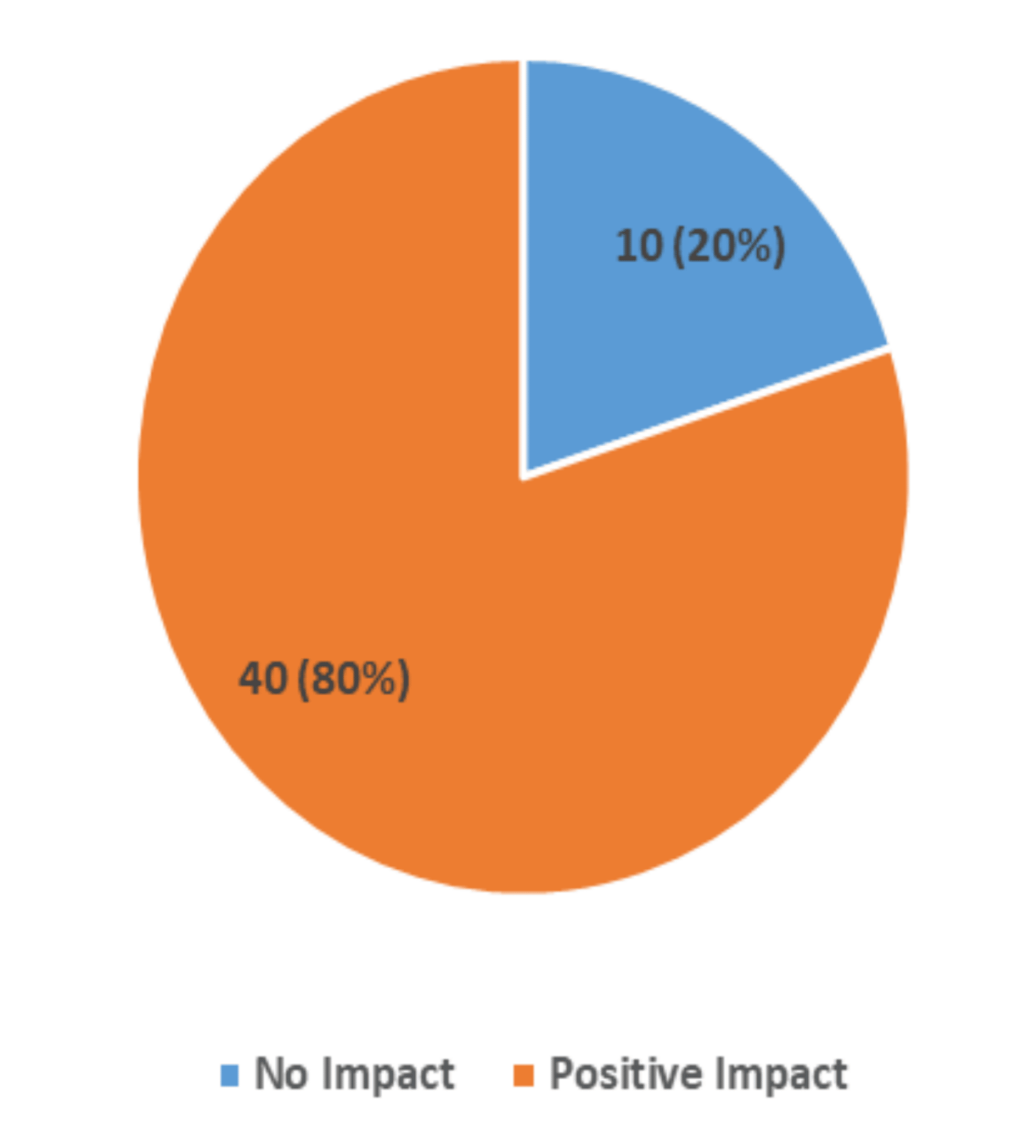
Impact of Home PC Access on Health Appointments and Information (50 Respondents)

**Figure 4:**
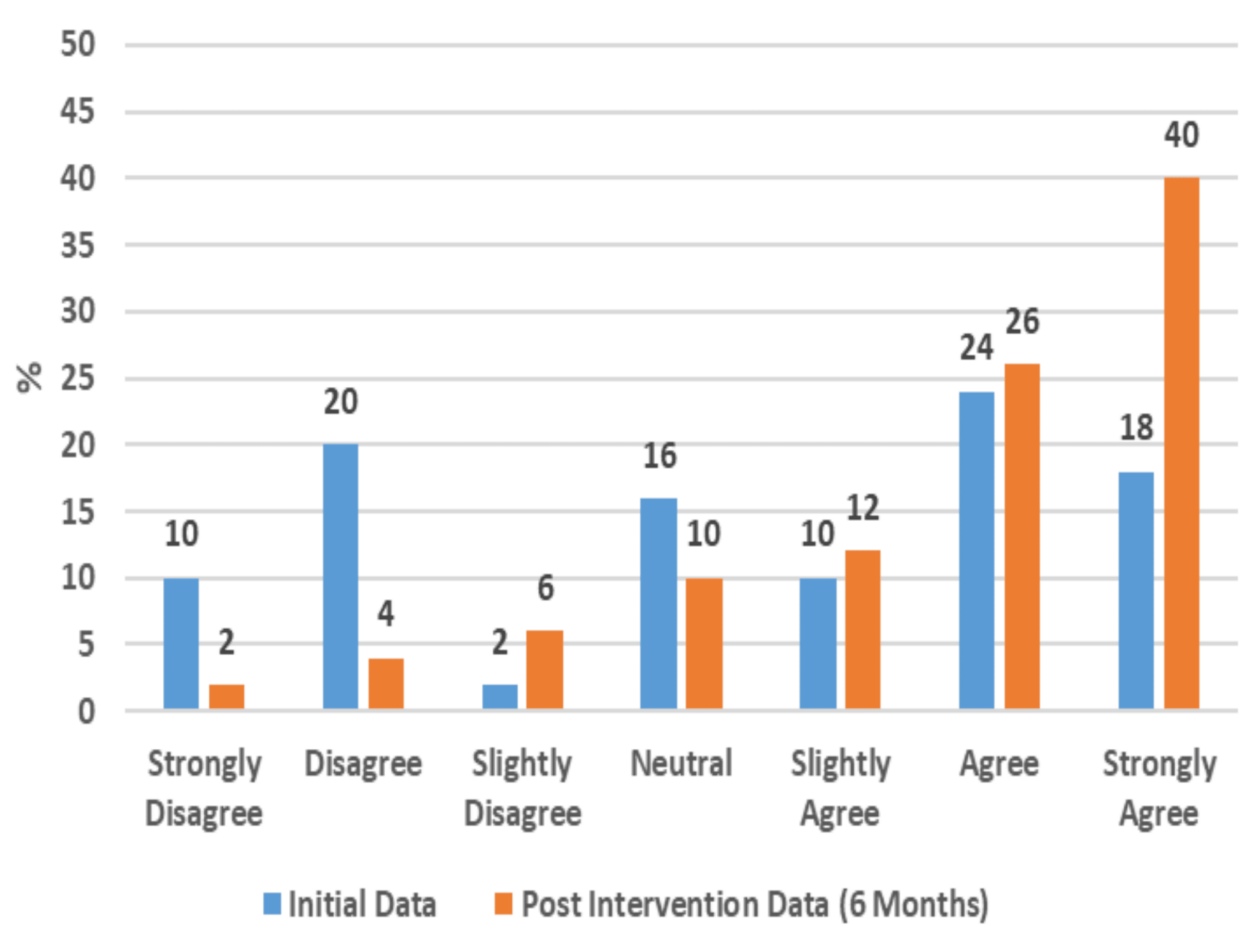
Perception That Health Was Risked Less by Being Able to Order Goods, Services, and Appointments From Home (50 Respondents)

The results also showcased strong positive impacts on participants' physical well-being, as evidenced by their newly gained ability to access health appointments and information from home (Figure 3) and by the reduced risk to their health from being able to order goods and services from home (Figure 4).

**Figure 5:**
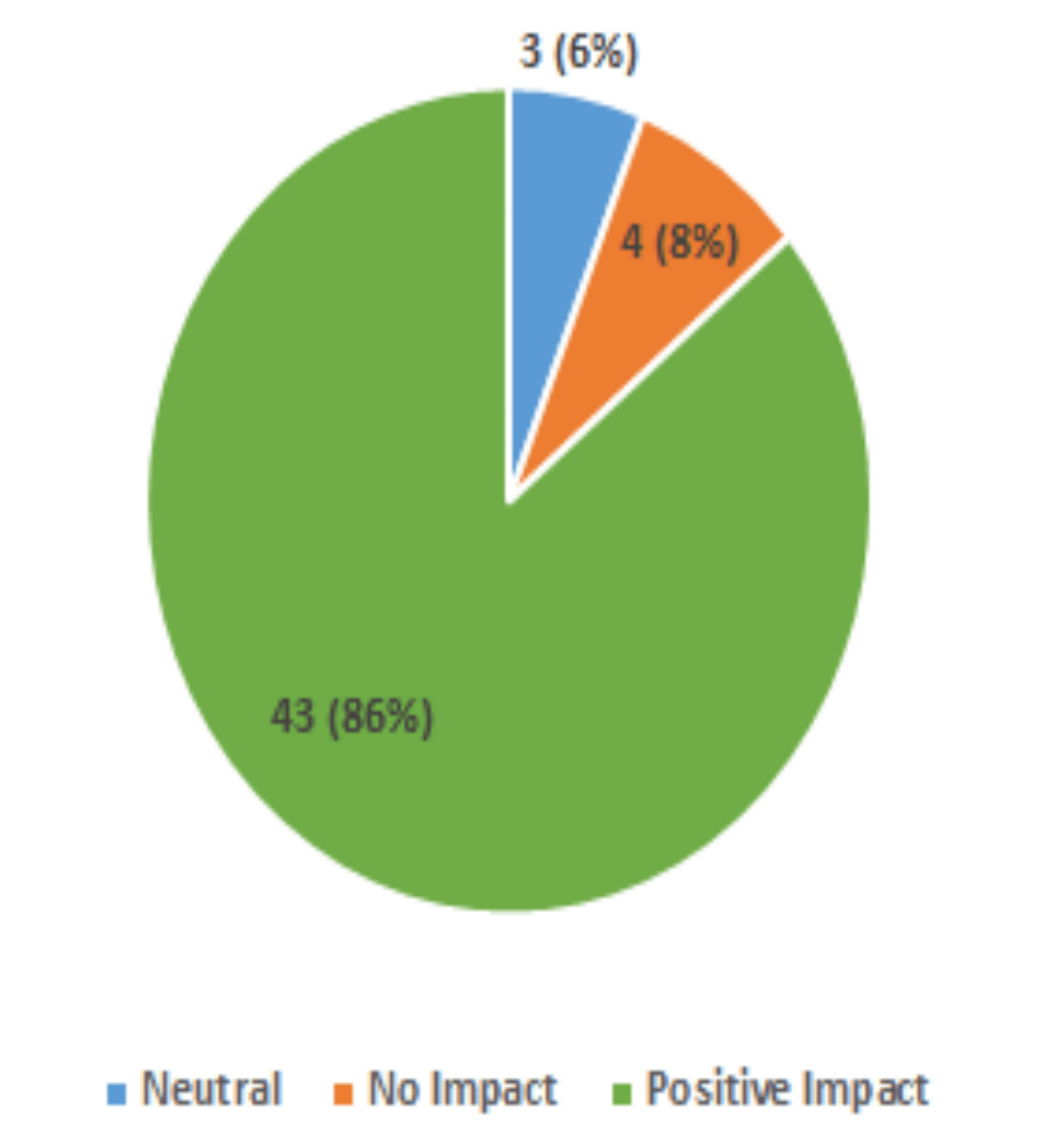
Impact of Home PC Access on Communication With Others (50 Respondents)

**Figure 6:**
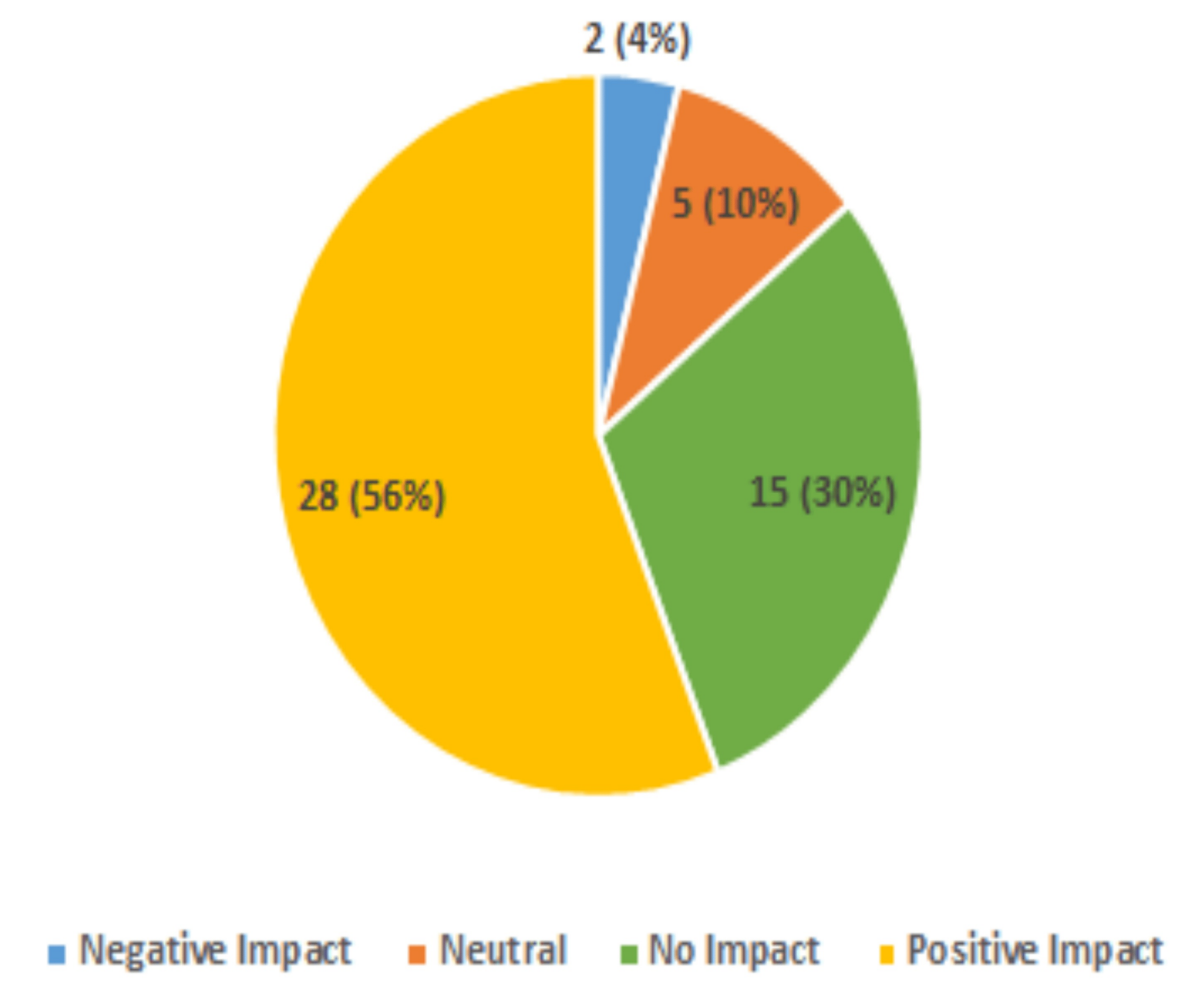
Impact of Home PC Access on Feelings of Isolation (50 Respondents)

**Figure 7:**
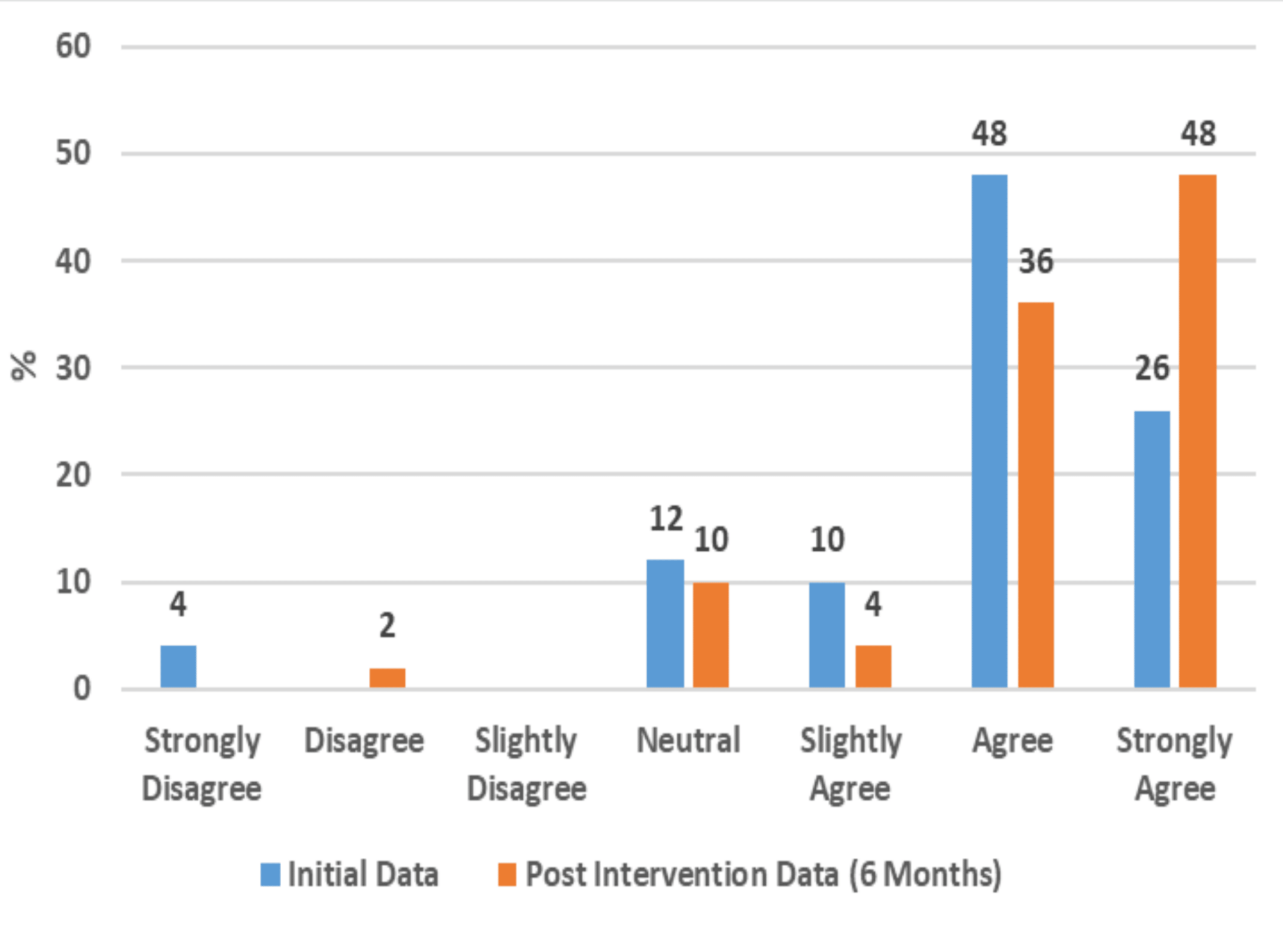
Perception That PC Will Help Connect With Others (50 Respondents)

This study further found remarkable positive impacts on participants' perceived social well-being. In total, 86% of the participants reported that their communication with others was positively impacted by having access to a PC and internet at home (Figure 5). This is also supported by 56% reporting that their PC had a positive impact on their feelings of isolation (Figure 6). Additionally, 48% now strongly agree and 26% agree that having PC access will help them connect with others (Figure 7).

**Figure 8:**
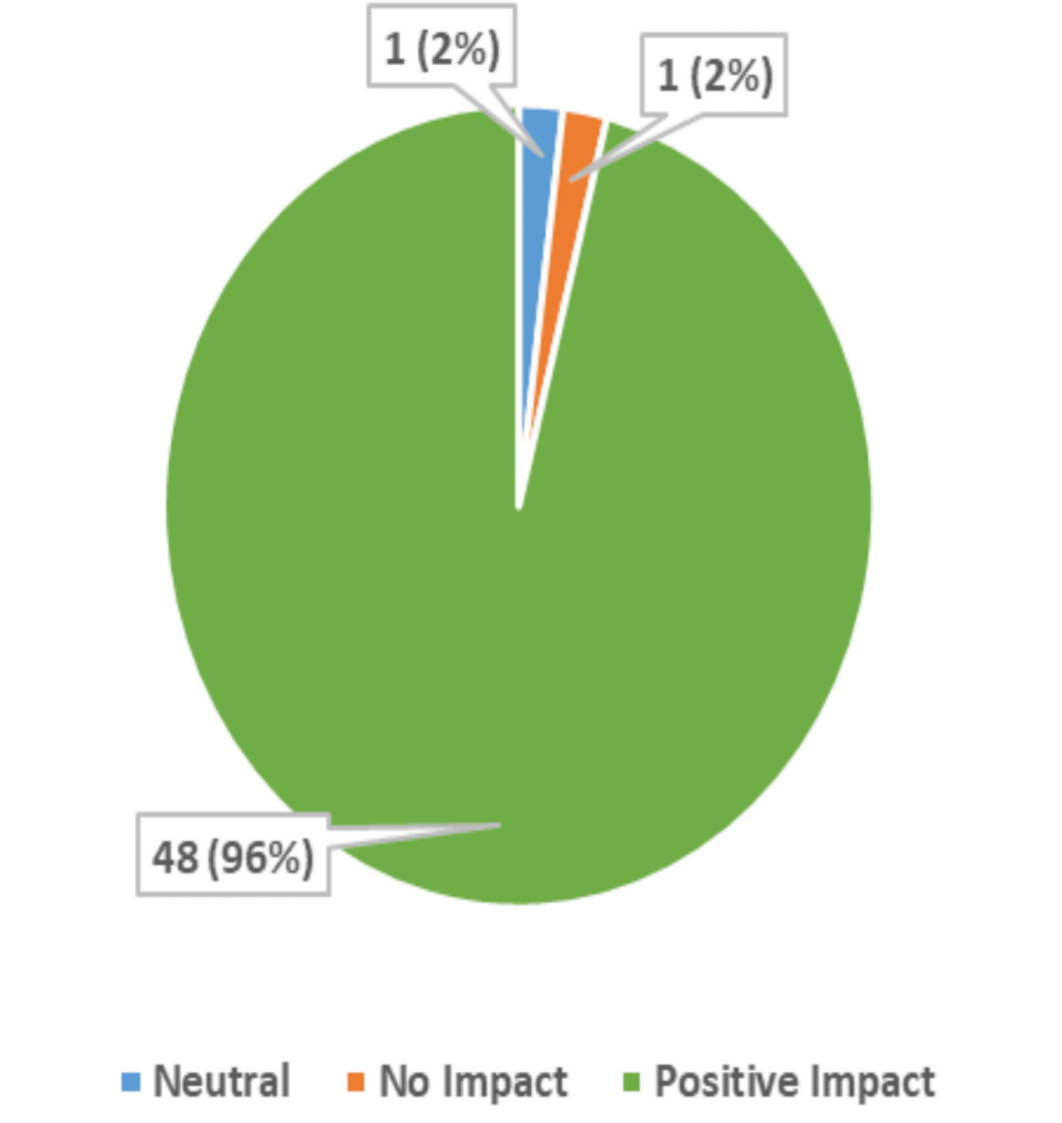
Impact of Home PC Access on Opportunities (50 Respondents)

**Figure 9:**
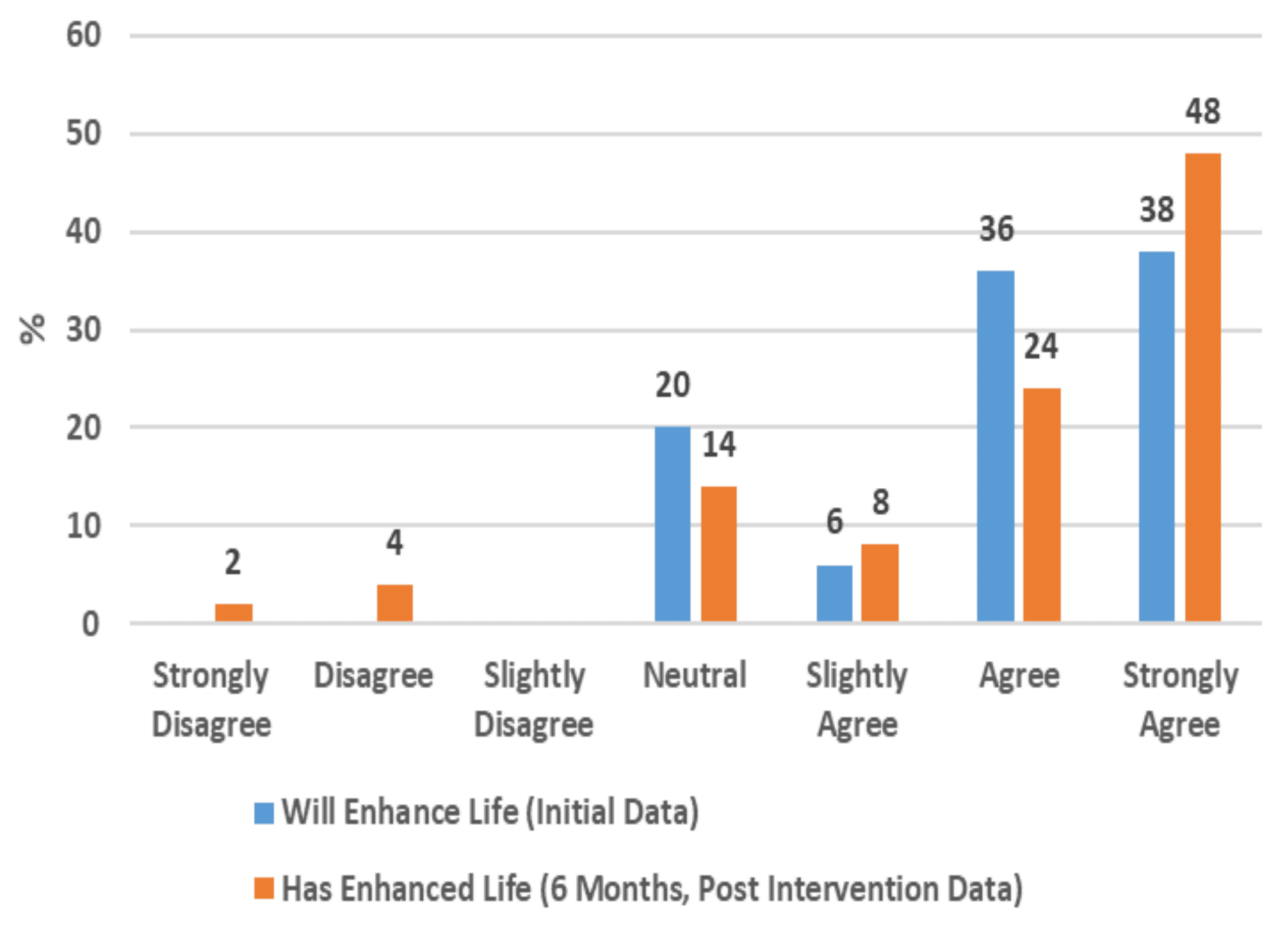
Perception of PC Enhances Quality of Life (50 Respondents)

**Figure 10:**
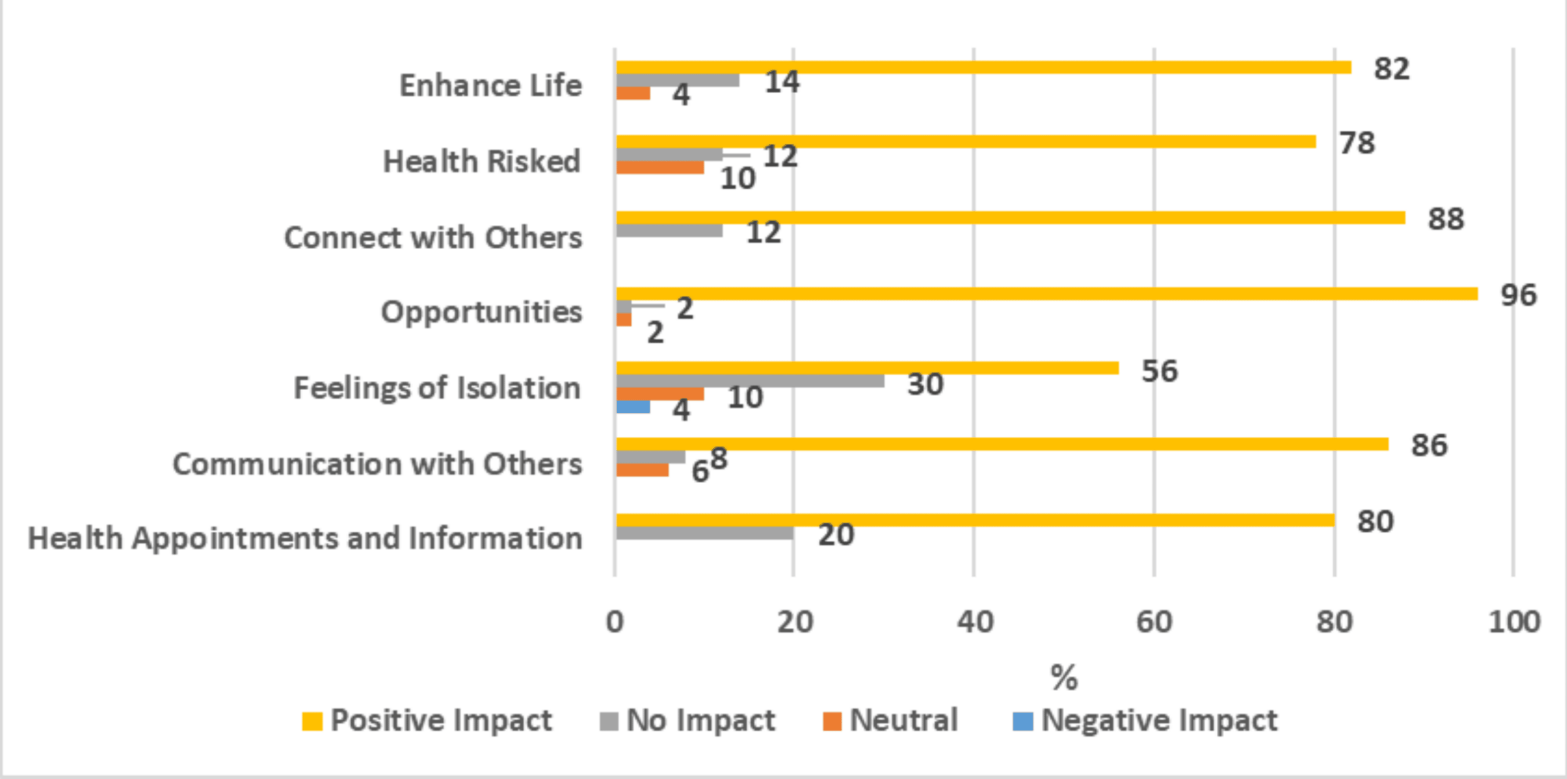
Overall Impact of PC Access at Home (50 Respondents)

For participants' general well-being, they report immense positive impacts of PC access on their opportunities (Figure 8) and on enhancing their lives (Figure 9). In summary, the data revealed that a clear majority of participants reported that having PC and internet access at home had a strong positive impact on their lives (Figure 10).

## Discussion

This study found that computers and reliable internet access had a strong, positive impact on participants' perceptions of their quality of life across multiple domains. After participating in this initiative, many participants showed an increase in perceived value of having access to a computer at home when exposed to a more comprehensive device and internet connection than their previous smartphone, tablet, or no device. Participants believed that the technology facilitated their economic progress by providing greater professional freedom. In fact, participants expressed a greater opportunity to work from home, which helped those who were physically unable to leave their house and, therefore, could not previously hold a job. Participants working from home could also be at home when their children returned from school, work remotely alongside their children while they learn remotely, and assist their children with school assignments. These positive changes highlight the importance of technology for low socioeconomic status groups, as 72% of the study's participants self-reported that their household income was less than $20,000, 25% reported making $20,000-$44,999, and 3% reported that their household income was between $45,000 and $139,999 annually.

Participants also expressed a positive impact on their social and emotional health as well by having an easier means to communicate and connect with their community. This finding supports that of Wellman et al. (2003), which found the internet to have "potential for social connectedness due to its social affordances, that is, the convenience, connectivity, and social cues that create opportunities for diverse interaction" [16].

As previously mentioned, this digital divide continues to widen between low and high socioeconomic status groups and among historically marginalized populations. With the higher number of Black and Hispanic groups having less access, specifically to computers and broadband connections [11], this program and our study helped bridge that divide. Of the study's participants, 87% identified as Black/African American, 7% identified as Other, and 6% identified as White. The participants' responses in the initial survey were representative of many of the obstacles that previous literature has revealed.

In slight contrast, a few participants reported some feelings of discomfort when using their computer and connecting with others. In further research, it may be beneficial to provide education and assistance in operating the computer and its systems to help alleviate those scores [17,18]. Pairing technology and internet access with user education may provide the most benefit overall for both the participant and study response rates [19].

Additionally, a potential benefit to this study and future studies with programs like this for institutions is an increase in their perceived commitment to their community. Grande et al. (2013) found programs like this not only benefit their community members but also bolster trust and loyalty to the institution from their community [20].

Furthermore, the researchers faced some limitations in conducting an online survey compared to face-to-face interviews. This study relied on the technology given to communicate with the participants. Researchers found that some individuals had complications with their emails or mistyped the email addresses during signup. Therefore, researchers had some difficulties contacting those individuals who elected to participate in the follow-up surveys. Researchers ultimately turned to texting the surveys and calling the individuals with the phone numbers that the participants provided, but responses were still somewhat difficult to obtain. With these participants not visiting the clinic regularly, the hope was that online surveys would be convenient and less time-consuming, overall a better experience for participants.

Additionally, the questionnaires administered to participants were not formally validated through expert panel review or pilot testing, which may affect the reliability and generalizability of our behavioral and attitudinal findings regarding participants' technology experiences. Future research may want to establish a face-to-face alternative for participants to gather more responses and address any questions they may have about the survey questions and the technology provided. Additionally, it would be interesting to do an extended longitudinal follow-up with this cohort and see if the benefits of the Rainbow Connect Program and supplied internet-connected devices extend beyond the six-month time frame of the initial intervention. This is being considered as a follow-up to the current study with longer time points.

Lastly, another limitation of this study may be its generalizability to the general population. This study was conducted in a metropolitan area where various public functions, such as religious meetings, grocery shopping, and government operations, for example, transitioned to online operations during the pandemic, and this area had the necessary resources and technology to support online functioning. This study and program helped to bridge the gap for individuals to access technology and the internet, enabling them to join the rest of their community. This may not translate to rural areas, whose needs to fit into a technology-dominant world are not as strong, as their communities may not have turned so heavily toward technology and online substitutes during the pandemic and thereafter.

## Conclusions

This study aimed to evaluate the perceived benefits of a computer and reliable internet access for disadvantaged households. The data revealed significant improvements in the overall perceived quality of life and in the mental, emotional, and physical well-being of the participants. This illustrates that connectivity programs like this are beneficial and can be crucial as a first step for institutions to promote digital connectivity in the communities they reside in and serve. As mentioned, programs like this benefit the community of institutions and showcase the institution's commitment to that community, which encourages more trust and loyalty from the community. The technology gap is real, but small interventions can have significant benefits for historically marginalized populations. This study further aims to encourage similar institutions to provide programs like this, continuing to aid in bridging the gap, as it is essential to keep individuals connected to their communities. More research is needed on the benefits of digital literacy training, specifically in terms of its ability to augment these programs and determine the longitudinal impact on participants' perceptions months or even years after the program is completed.

## Appendices

Supplemental Material 1. Initial Survey Instrument

PC follow-up survey identification number: ___________________________________

What is your name? ___________________________________

What is your email? ___________________________________

What is your phone number? ___________________________________

What is your gender? ___________________________________

What is your age? ___________________________________

What is your race?

o   White/Caucasian

o   Black/African American

o   Other

What is your zip code? ___________________________________

What is your highest level of education?

o   Less than a high school diploma

o   High school diploma or GED

o   Associate’s degree

o   Bachelor’s degree

o   Master’s degree

o   Professional degree

o   Doctorate

What is your household income range?

o   Less than $20,000

o   $20,000 - $44,999

o   $45,000 - $139,999

o   $140,000 - $149,999

o   $150,000 - $199,999

o   $200,000+

Is this a single household income?

o   Yes

o   No

How many individuals are in your household?

o   0

o   1

o   2

o   3

o   4

o   5

o   6

o   7

o   8

o   9

o   10

o   More than 10

How many of those individuals are children (under 18) in your household?

o   0

o   1

o   2

o   3

o   4

o   5

o   6

o   7

o   8

o   9

o   10

o   More than 10

What is your primary language spoken at home?

o   English

o   Spanish

o   Arabic

o   Chinese [Mandarin, Cantonese]

o   Russian [including Slavic and Serbo-Croatian rooted languages]

o   Indic [Hindi, Urdu, Indo-European]

o   French

o   Other language

Do you speak another language at home?

o   English

o   Spanish

o   Arabic

o   Chinese [Mandarin, Cantonese]

o   Russian [including Slavic and Serbo-Croatian rooted languages]

o   Indic [Hindi, Urdu, Indo-European]

o   French

o   Other language

o   None (no other language spoken)

The next section will tell us a little bit about your current computer and internet usage and access.

Have you ever used a computer?

o   Yes

o   No

Why was it hard or difficult for you to get a computer before today?

o   It cost too much

o   Didn’t know which computer to buy

o   Did not know how to use a computer

o   Didn’t know if I would need or use a computer

o   No internet access

o   No home or housing

o   Other

Other Barriers (please explain) ___________________________________

How comfortable do you feel using the computer?

o   1. Very Uncomfortable

o   2. Uncomfortable

o   3. Slightly Uncomfortable

o   4. Neutral

o   5. Slightly Comfortable

o   6. Comfortable

o   7. Very Comfortable

Have you owned a computer before?

o   Yes

o   No

Do you currently have a computer in your home or a computer that you use at home?

o   Yes

o   No

If no, is this due to financial reasons or personal preference?

o   Financial

o   Preference

Do you have access to the internet at home? (not by using your cell-phone)

o   Yes- Broadband/Highspeed

o   Yes- Hotspot

o   Yes- Other

o   No

Can you access your email at home?

o   Yes

o   No

Do you have access to the internet or email at home through other means (for example, a smartphone or tablet)?

o   Yes

o   No

Would you like Rainbow Connect to contact you about our Computer Connection Program?

o   Yes

o   No

The next section will tell us a little about your feelings involving technology.

Has not having a computer at home made it difficult for you to access health appointments or health information?

o   1. Strongly Disagree

o   2. Disagree

o   3. Slightly Disagree

o   4. Neutral

o   5.. Slightly Agree

o   6. Agree

o   7. Strongly Agree

Has not having a computer at home made it difficult for you to communicate with others?

o   Yes

o   No

o   NA

Has not having a computer at home made you feel isolated from others?

o   1. Strongly Disagree; Not feeling isolated at all

o   2. Disagree

o   3. Slightly Disagree

o   4. Neutral

o   5. Slightly Agree

o   6. Agree

o   7. Strongly Agree; Feeling strongly isolated

Do you feel as though you have missed opportunities due to not having a computer?

o   1. Strongly Disagree

o   2. Disagree

o   3. Slightly Disagree

o   4. Neutral

o   5. Slightly Agree

o   6. Agree

o   7. Strongly Agree

Do you feel as though this computer will help you connect to others?

o   1. Strongly Disagree

o   2. Disagree

o   3. Slightly Disagree

o   4. Neutral

o   5. Slightly Agree

o   6. Agree

o   7. Strongly Agree

Do you feel it is important to own a computer?

o   1. Strongly Disagree

o   2. Disagree

o   3. Slightly Disagree

o   4. Neutral

o   5. Slightly Agree

o   6. Agree

o   7. Strongly Agree

Do you feel that your health was risked less by having a computer at home to be able to order goods/services sent to your home (for example, groceries, clothing, appointments, etc.) during the COVID-19 pandemic, rather than having to leave your home to access these things?

o   1. Strongly Disagree

o   2. Disagree

o   3. Slightly Disagree

o   4. Neutral

o   5. Slightly Agree

o   6. Agree

o   7. Strongly Agree

Do you feel that having a computer at home will enhance your life?

o   1. Strongly Disagree

o   2. Disagree

o   3. Slightly Disagree

o   4. Neutral

o   5. Slightly Agree

o   6. Agree

o   7. Strongly Agree

Supplemental Material 2. Follow-up Survey Instrument

PC follow-up survey

The purpose of this survey is to know a little bit about the background of the population we are serving through this initiative.

This survey will take less than 2 minutes to complete.

You will receive a $5.00 Starbucks gift card after finishing the survey.

PC Identification Number ___________________________________

Do you have access to the internet at home?

o   Yes

o   No

Do you feel comfortable using your computer?

o   Yes

o   No

Can you access your email at home?

o   Yes

o   No

Do you have access to the internet or email at home through other means (for example, a smartphone or tablet)?

o   Yes

o   No

How comfortable do you feel using the computer?

o   1. Very Uncomfortable

o   2. Uncomfortable

o   3. Slightly Uncomfortable

o   4. Neutral

o   5. Slightly Comfortable

o   6. Comfortable

o   7. Very Comfortable

This next section will tell us a little about your feelings involving technology.

Has having a computer at home made it easier for you to access health appointments or health information?

o   1. Strongly Disagree

o   2. Disagree

o   3. Slightly Disagree

o   4. Neutral

o   5. Slightly Agree

o   6. Agree

o   7. Strongly Agree

Has having a computer at home made it easier for you to communicate with others?

o   Yes

o   No

o   NA

Has having a computer at home made you feel less isolated from others?

o   1. Strongly Disagree; Not feeling isolated at all

o   2. Disagree

o   3. Slightly disagree

o   4. Neutral

o   5. Slightly Agree

o   6. Agree

o   7. Strongly Agree; Feeling strongly Isolated

Do you feel you have more opportunities by having a computer and internet at home?

o   1. Strongly Disagree

o   2. Disagree

o   3. Slightly Disagree

o   4. Neutral

o   5. Slightly Agree

o   6. Agree

o   7. Strongly Agree

Has this computer helped you to connect with others?

o   1. Strongly Disagree

o   2. Disagree

o   3. Slightly Disagree

o   4. Neutral

o   5. Slightly Agree

o   6. Agree

o   7. Strongly Agree

Do you feel it is important to own a computer?

o   1. Strongly Disagree

o   2. Disagree

o   3. Slightly Disagree

o   4. Neutral

o   5. Slightly Agree

o   6. Agree

o   7. Strongly Agree

Do you feel that your health was risked less by having a computer at home to be able to order goods/services sent to your home (for example, groceries, clothing, appointments, etc.) during the COVID-19 pandemic, rather than having to leave your home to access these things?

o   1. Strongly Disagree

o   2. Disagree

o   3. Slightly Disagree

o   4. Neutral

o   5. Slightly Agree

o   6. Agree

o   7. Strongly Agree

Do you feel that having a computer at home has enhanced your life?

o   1. Strongly Disagree

o   2. Disagree

o   3. Slightly Disagree

o   4. Neutral

o   5. Slightly Agree

o   6. Agree

o   7. Strongly Agree

Supplemental Material 3. Demographics of All Participants

**Table 2:** Demographics of All 100 Participants

Questions	Levels	Overall, n = 100
What is your gender? (%)	Female	94 (94.0)
	Male	6 (6.0)
What is your age? (median [IQR])	-	31.00 [27.00, 37.00]
What is your race? (%)	Black/African American	87 (87.0)
	Other	7 (7.0)
	White/Caucasian	6 (6.0)
What is your highest level of education? (%)	Associate's degree	11 (11.0)
	Bachelor's degree	2 (2.0)
	High school diploma or GED	74 (74.0)
	Less than high school diploma	12 (12.0)
	Professional degree	1 (1.0)
What is your household income range? (%)	$20,000 - $44,999	25 (25.0)
	$45,000 - $139,999	3 (3.0)
	Less than $20,000	72 (72.0)
Is this a single household income? (%)	No	8 (8.0)
	Yes	92 (92.0)
How many individuals are in your household? (median [IQR])	-	3.00 [2.00, 4.00]
How many of those individuals are children under 18 in your household? (median [IQR])	-	2.00 [1.00, 3.00]
What is your primary language spoken at home? (%)	English	100 (100.0)
Do you speak another language at home? (%)	Arabic	1 (1.0)
	None (No other language spoken)	93 (93.0)
	Other language	1 (1.0)
	Spanish	5 (5.0)
Have you ever used a computer? (%)	No	7 (7.0)
	Yes	93 (93.0)
Why was it hard or difficult for you to get a computer before today? (%)	Didn't know if I would need or use a computer	5 (5.0)
	Didn't know which computer to buy	4 (4.0)
	It cost too much	80 (80.0)
	No internet access	3 (3.0)
	Other	8 (8.0)
Other barriers please explain. (%) [free text]	It cost too much, couldn't connect to wifi	1 (20.0)
	Need another computer for child use mines not working properly	1 (20.0)
	No problem at all	1 (20.0)
	Not difficult	1 (20.0)
	not hard	1 (20.0)
How comfortable do you feel using the computer? (%)	1. Very Uncomfortable	39 (39.0)
	2. Uncomfortable	2 (2.0)
	3. Slightly Uncomfortable	2 (2.0)
	4. Neutral	7 (7.0)
	5. Slightly Comfortable	5 (5.0)
	6. Comfortable	23 (23.0)
	7. Very Comfortable	22 (22.0)
Have you owned a computer before? (%)	No	45 (45.0)
	Yes	55 (55.0)
Do you currently have a computer in your home or a computer that you use at home? (%)	No	88 (88.0)
	Yes	12 (12.0)
If no, is this due to financial reasons or personal preference? (%)	Financial	74 (84.1)
	Preference	14 (15.9)
Do you have access to internet at home not by using your cell phone? (%)	No	45 (45.0)
	Yes- Broadband/Highspeed	31 (31.0)
	Yes- Hotspot	16 (16.0)
	Yes- Other	8 (8.0)
Can you access your email at home? (%)	No	8 (8.0)
	Yes	92 (92.0)
Do you have access to internet or email at home through other means, for example smartphone or tablet? (%)	No	12 (12.0)
	Yes	88 (88.0)
Would you like rainbow connect to contact you about our computer connection program? (%)	No	22 (22.0)
	Yes	78 (78.0)
Has not having a computer at home made it difficult for you to access health appointments or health information? (%)	1. Strongly Disagree	11 (11.0)
	2. Disagree	11 (11.0)
	3. Slightly Disagree	4 (4.0)
	4. Neutral	23 (23.0)
	5. Slightly Agree	16 (16.0)
	6. Agree	18 (18.0)
	7. Strongly Agree	17 (17.0)
Has not having a computer at home made it difficult for you to communicate with others? (%)	NA	15 (15.0)
	No	44 (44.0)
	Yes	41 (41.0)
Has not having a computer at home made you feel isolated from others? (%)	1. Strongly Disagree; Not feeling isolated at all	11 (11.0)
	2. Disagree	24 (24.0)
	3. Slightly Disagree	7 (7.0)
	4. Neutral	23 (23.0)
	5. Slightly Agree	15 (15.0)
	6. Agree	13 (13.0)
	7. Strongly Agree; Feeling strongly isolated	7 (7.0)
Do you feel as though you have missed opportunities due to not having a computer? (%)	1. Strongly Disagree	5 (5.0)
	2. Disagree	6 (6.0)
	3. Slightly Disagree	2 (2.0)
	4. Neutral	17 (17.0)
	5. Slightly Agree	17 (17.0)
	6. Agree	28 (28.0)
	7. Strongly Agree	25 (25.0)
Do you feel as though this computer will help you connect to others? (%)	1. Strongly Disagree	3 (3.0)
	2. Disagree	1 (1.0)
	3. Slightly Disagree	2 (2.0)
	4. Neutral	12 (12.0)
	5. Slightly Agree	7 (7.0)
	6. Agree	43 (43.0)
	7. Strongly Agree	32 (32.0)
Do you feel it is important to own a computer? (%)	1. Strongly Disagree	2 (2.0)
	4. Neutral	11 (11.0)
	5. Slightly Agree	3 (3.0)
	6. Agree	34 (34.0)
	7. Strongly Agree	50 (50.0)
Do you feel your health was risked by having to leave your home due to not having a computer to be able to order goods services sent to your home during the covid 19 pandemic quarantine mandates (for example; groceries, clothing, appointments, etc.)? (%)	1. Strongly Disagree	7 (7.0)
	2. Disagree	19 (19.0)
	3. Slightly Disagree	2 (2.0)
	4. Neutral	19 (19.0)
	5. Slightly Agree	11 (11.0)
	6. Agree	25 (25.0)
	7. Strongly Agree	17 (17.0)
Do you feel that having a computer at home will enhance your life? (%)	1. Strongly Disagree	1 (1.0)
	2. Disagree	2 (2.0)
	3. Slightly Disagree	1 (1.0)
	4. Neutral	13 (13.0)
	5. Slightly Agree	7 (7.0)
	6. Agree	35 (35.0)
	7. Strongly Agree	41 (41.0)
County (%)	Ashtabula County	1 (1.0)
	Cuyahoga County	95 (96.0)
	Fayette County	1 (1.0)
	Geauga County	1 (1.0)
	Summit County	1 (1.0)
Would you like to be contacted for a follow-up survey? (%)	No	14 (14.0)
	Yes	86 (86.0)

Supplemental Material 4. Responses From 50 Participants at Six Months

**Table 3:** Responses From 50 Participants at Six Months

Questions	Levels	Overall, n = 50
Do you feel confident using your computer? (%)	Yes	50 (100.0)
How comfortable do you feel using the computer? (%)	1. Very uncomfortable	5 (10.0)
	3. Slightly uncomfortable	1 (2.0)
	4. Neutral	4 (8.0)
	5. Slightly comfortable	2 (4.0)
	6. Comfortable	7 (14.0)
	7. Very comfortable	31 (62.0)
How comfortable do you feel using the computer? [Numeric Likert] (median [IQR])	-	7.00 [6.00, 7.00]
Do you have access to the internet at home? (%)	No	6 (12.0)
	Yes	44 (88.0)
Can you access your email at home? (%)	No	2 (4.0)
	Yes	48 (96.0)
Do you have access to the internet or email at home through other means, for example, a smartphone or tablet? (%)	No	1 (2.0)
	Yes	49 (98.0)
Has having a computer at home made it easier for you to access health appointments or health information? (%)	1. Strongly Disagree	1 (2.0)
	2. Disagree	1 (2.0)
	3. Slightly Disagree	2 (4.0)
	4. Neutral	6 (12.0)
	5. Slightly Agree	3 (6.0)
	6. Agree	11 (22.0)
	7. Strongly Agree	26 (52.0)
Has having a computer at home made it easier for you to communicate with others? (%)	NA	3 (6.0)
	No	4 (8.0)
	Yes	43 (86.0)
Has having a computer at home made you feel less isolated from others? (%)	1. Strongly Disagree; Not feeling isolated at all	7 (14.0)
	2. Disagree	6 (12.0)
	3. Slightly Disagree	1 (2.0)
	4. Neutral	8 (16.0)
	5. Slightly Agree	4 (8.0)
	6. Agree	8 (16.0)
	7. Strongly Agree	16 (32.0)
Do you feel you have more opportunities by having a computer and internet at home? (%)	3. Slightly Disagree	1 (2.0)
	4. Neutral	3 (6.0)
	5. Slightly Agree	2 (4.0)
	6. Agree	14 (28.0)
	7. Strongly Agree	30 (60.0)
Has this computer helped you connect to others? (%)	2. Disagree	1 (2.0)
	4. Neutral	5 (10.0)
	5. Slightly Agree	2 (4.0)
	6. Agree	18 (36.0)
	7. Strongly Agree	24 (48.0)
Do you feel it is important to own a computer? (%)	4. Neutral	1 (2.0)
	5. Slightly Agree	2 (4.0)
	6. Agree	14 (28.0)
	7. Strongly Agree	33 (66.0)
Do you feel your health was risked by having to leave your home due to not having a computer to be able to order goods services sent to your home during the COVID-19 pandemic quarantine mandates (for example, groceries, clothing, appointments, etc.)? (%)	1. Strongly Disagree	1 (2.0)
	2. Disagree	2 (4.0)
	3. Slightly Disagree	3 (6.0)
	4. Neutral	5 (10.0)
	5. Slightly Agree	6 (12.0)
	6. Agree	13 (26.0)
	7. Strongly Agree	20 (40.0)
Do you feel that having a computer at home has enhanced your life? (%)	1. Strongly Disagree	1 (2.0)
	2. Disagree	2 (4.0)
	4. Neutral	7 (14.0)
	5. Slightly Agree	4 (8.0)
	6. Agree	12 (24.0)
	7. Strongly Agree	24 (48.0)

Supplemental Material 5. Overall Changes and Impact on Perceptions From Baseline to Six Months

**Table 4:** Overall Changes and Impact on Perceptions From Baseline to Six Months

Questions	level	Overall, n = 50
Change: How comfortable do you feel using the computer? (%)	From comfortable to neutral	3 (6.0)
	From comfortable to uncomfortable	2 (4.0)
	From uncomfortable/neutral to comfortable	21 (42.0)
	Still comfortable	24 (48.0)
Change: How comfortable do you feel using the computer? [Numeric Likert] (median [IQR])	-	0.50 [0.00, 3.00]
Gained access to the internet at home. (%)	No	3 (15.0)
	Yes	17 (85.0)
Gained access to email at home. (%)	Yes	6 (100.0)
Gained access to the internet or email at home through other means. (%)	Yes	6 (100.0)
Impact of having a computer at home in access health appointments or health information. (%)	No impact	10 (20.0)
	Positive impact	40 (80.0)
Impact of having a computer at home and communication with others. (%)	Neutral	3 (6.0)
	No Impact	4 (8.0)
	Positive Impact	43 (86.0)
Impact of having a computer at home and feeling less isolated from others. (%)	Negative impact	2 (4.0)
	Neutral	5 (10.0)
	No impact	15 (30.0)
	Positive Impact	28 (56.0)
Impact of having more opportunities by having a computer and the internet at home. (%)	Neutral	1 (2.0)
	No Impact	1 (2.0)
	Positive Impact	48 (96.0)
Impact on the computer helping the recipient connect to others. (%)	No impact	6 (12.0)
	Positive Impact	44 (88.0)
Change: Do you feel it is important to own a computer? (%)	Improved	7 (14.0)
	Neutral	1 (2.0)
	Still agrees is important	42 (84.0)
Impact on: Do feel your health is risked less by having a computer at home to be able to order goods services sent to your home? (%)	Neutral	5 (10.0)
	No impact	6 (12.0)
	Positive impact	39 (78.0)
Impact on: Do you feel that having a computer at home has enhanced your life? (%)	Neutral	2 (4.0)
	No Impact	7 (14.0)
	Positive impact	4 (8.0)
	Positive Impact	37 (74.0)
